# Comparative quantitative systems pharmacology modeling of anti-PCSK9 therapeutic modalities in hypercholesterolemia[S]

**DOI:** 10.1194/jlr.M092486

**Published:** 2019-07-10

**Authors:** Victor Sokolov, Gabriel Helmlinger, Catarina Nilsson, Kirill Zhudenkov, Stanko Skrtic, Bengt Hamrén, Kirill Peskov, Eva Hurt-Camejo, Rasmus Jansson-Löfmark

**Affiliations:** M&S Decisions,* Moscow, Russia; Clinical Pharmacology & Safety Sciences† R&D BioPharmaceuticals, AstraZeneca, Boston, MA; Clinical Pharmacology & Safety Sciences§ R&D BioPharmaceuticals, AstraZeneca, Gothenburg, Sweden; Cardiovascular, Renal and Metabolism§§ R&D BioPharmaceuticals, AstraZeneca, Gothenburg, Sweden; Institute of Medicine at Sahlgrenska Academy,** University of Gothenburg, Gothenburg, Sweden; I. M. Sechenov First Moscow State Medical University of the Russian Ministry of Health†† Moscow, Russia

**Keywords:** atherosclerosis, lipoproteins, cholesterol metabolism, plasma proprotein convertase subtilisin/kexin type 9, quantitative modeling, translational pharmacology

## Abstract

Since the discovery of proprotein convertase subtilisin/kexin type 9 (PCSK9) as an attractive target in the treatment of hypercholesterolemia, multiple anti-PCSK9 therapeutic modalities have been pursued in drug development. The objective of this research is to set the stage for the quantitative benchmarking of two anti-PCSK9 pharmacological modality classes, monoclonal antibodies (mAbs) and small interfering RNA (siRNA). To this end, we developed an integrative mathematical model of lipoprotein homeostasis describing the dynamic interplay between PCSK9, LDL-cholesterol (LDL-C), VLDL-cholesterol, HDL-cholesterol (HDL-C), apoB, lipoprotein a [Lp(a)], and triglycerides (TGs). We demonstrate that LDL-C decreased proportionally to PCSK9 reduction for both mAb and siRNA modalities. At marketed doses, however, treatment with mAbs resulted in an additional ∼20% LDL-C reduction compared with siRNA. We further used the model as an evaluation tool and determined that no quantitative differences were observed in HDL-C, Lp(a), TG, or apoB responses, suggesting that the disruption of PCSK9 synthesis would provide no additional effects on lipoprotein-related biomarkers in the patient segment investigated. Predictive model simulations further indicate that siRNA therapies may reach reductions in LDL-C levels comparable to those achieved with mAbs if the current threshold of 80% PCSK9 inhibition via siRNA could be overcome.

Atherosclerotic cardiovascular disease (ACVD) is the leading cause of death in developed countries, with hypercholesterolemia being the main risk factor for disease progression (1). Epidemiological studies show that the risk for ACVD mortality is correlated with the number of apoB lipoprotein particles, clinically assessed through measurements of plasma apoB, non-HDL-cholesterol (HDL-C), and LDL-cholesterol (LDL-C) (2). Different yet complementary means, including lifestyle changes, diet, and medications, may be used to achieve desirable cholesterol levels. Statins are safe and efficacious drugs for reducing levels of apoB lipoproteins and consequently for decreasing the risk of ACVD clinical events in most patients (3). However, in patients with inherited disorders that result in high plasma LDL-C levels, treatment with statins do not always reach sufficiently low LDL-C levels (e.g., <70 mg/dl), even in combination with ezetimibe, a cholesterol uptake inhibitor (4). Additionally, some patients cannot be maintained on adequate doses of statins due to adverse events such as myopathy (5).

The discovery of the proprotein convertase subtilisin/kexin type 9 (PCSK9) and the protein’s ability to regulate LDL-receptor (LDL-R) numbers expressed on hepatocytes made PCSK9 a potent target against hypercholesterolemia and resulted in a drug-development surge of various anti-PCSK9 pharmacological modalities (6). As many as nine different therapies are currently in development or on the market: monoclonal antibodies (mAbs), GLP1-fused antibodies, antibody mimetics, small molecules, siRNA, antisense oligonucleotides, peptide vaccines against self-antigens, as well as CRISPR approaches (7). Among these, two classes of anti-PCSK9 compounds have been shown to effectively decrease plasma LDL-C in humans: mAbs, which bind PCSK9 and remove it from the systemic circulation, and siRNA, which blocks the intracellular synthesis of PCSK9, mainly in hepatocytes. Despite sharing the same target, these two modalities exert their effects through differing mechanisms of action and pharmacokinetic profiles, as reflected in longitudinal measurements of multiple clinical biomarkers.

In this work, we sought to compare the efficacy and related biomarker profiles for the mAb and siRNA modalities in a common, integrative, and quantitative modeling framework of lipoprotein metabolism. Several mechanistic quantitative systems pharmacology (QSP) models have been described in the literature, covering various aspects of lipoprotein metabolism (8–11). Here, from an intricate spectrum of detailed lipoprotein metabolism processes, we derived a fit-for-purpose mechanistic model that includes key efficacy-related biomarkers. The model is, at the same time, adequately parameterized and calibrated, overcoming the usual challenges of variability, uncertainty, and missing data encountered in clinical studies (**Fig. 1**). It quantitatively characterizes and predicts the time courses of LDL-C, non-HDL-C, VLDL-cholesterol (VLDL-C), HDL-cholesterol (HDL-C), total cholesterol (TC), apoB, lipoprotein A [Lp(a)], and triglycerides (TGs) in response to five therapies (three mAbs: alirocumab, evolocumab, and RG-7652; and two siRNAs: inclisiran and ALN-PCS), for all dosing regimens of interest, in simulations of healthy subjects and hypercholesterolemia patients on statin treatment.

**Fig. 1.**
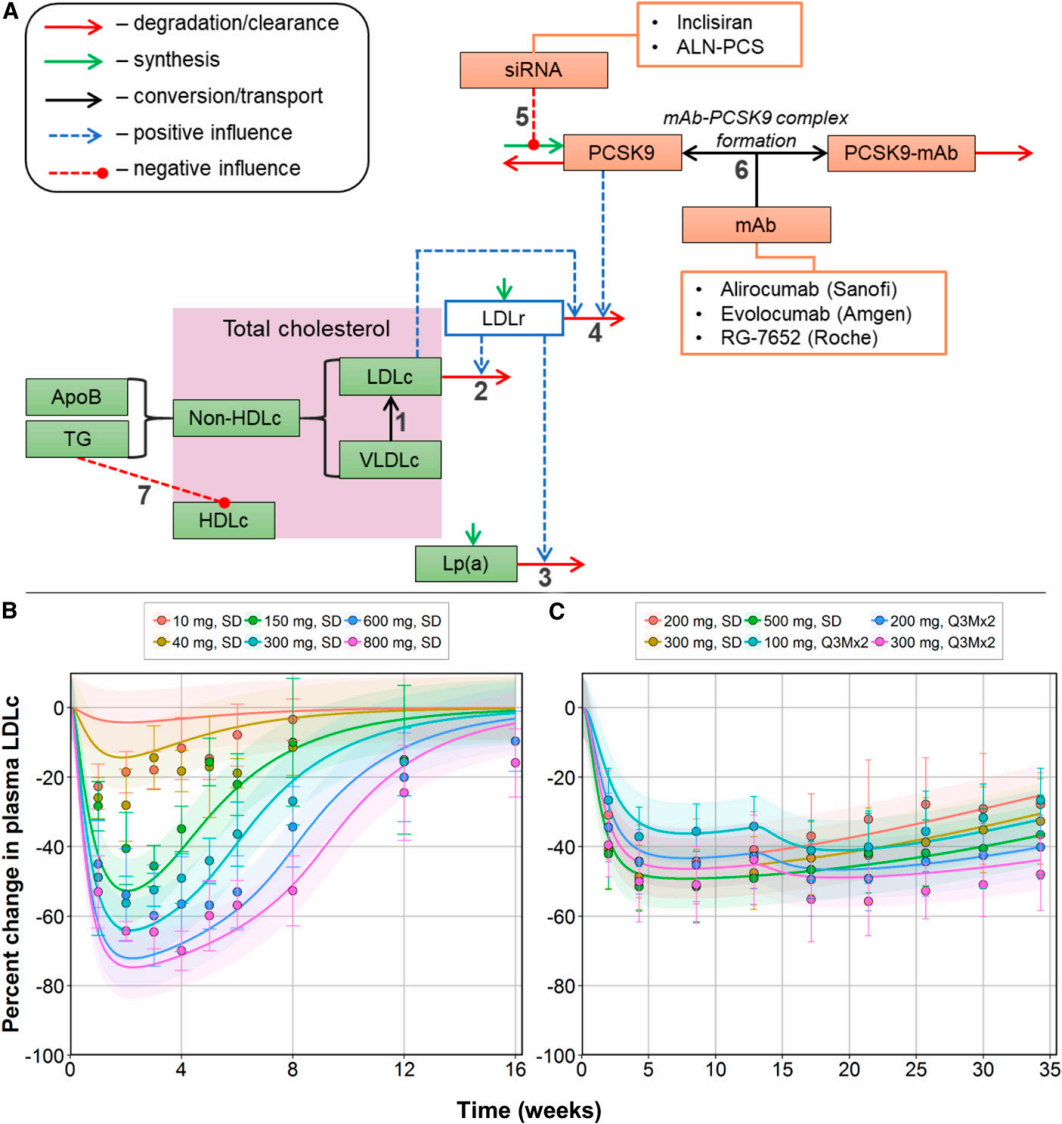
Structural elements and interactions captured in the mechanistic QSP model (A) and model predictions of mean plasma LDL-C dynamics under treatment with anti-PCSK9 mAbs (B) and siRNA (C). Comparisons of plasma LDL-C levels measured at weeks 0–16 for RG-7652 data and weeks 0–34 (colored dots with error bars; mean ± standard deviation) for inclisiran in clinical studies versus corresponding model-based simulations (line with shaded area; mean ± 95% confidence prediction interval of the model). All values were normalized to absolute LDL-C values from each dosing arm. Experimental data from (27, 30). Values are presented in percentage change from baseline.

## MATERIALS AND METHODS

### Summary of model development

The model development consisted of five main steps. Due to the unavailability of subject-level data in open-source publications, mean aggregated data from each dosing arm of each investigated trial were used for the model development. In a first step, the model was calibrated using mean experimental data of plasma pharmacokinetic (PK) profiles of alirocumab and evolocumab, as well as mean plasma PCSK9 and LDL-C time profiles under treatment with alirocumab and evolocumab (12–26). Data were integrated from a total of 14 phase 1/2 and 3 phase 3 clinical trials comprising 68 dosing arms in total. Subjects were randomly assigned to single or multiple dose administrations in 10 trials of alirocumab and 7 trials of evolocumab. Doses were in the 50–300 mg range for alirocumab and 7–420 mg range for evolocumab. Data were collected for treatment periods ranging from 0.5 to 24 weeks. Four trials involved healthy subjects, and 16 trials primarily enrolled familial or nonfamilial hypercholesterolemia patients. Approximately 70% of all hypercholesterolemia subjects also received statins as a standard of care in addition to the antibody.

In a second step, the model was calibrated using mean aggregated data from healthy and hypercholesterolemia subjects given either intravenous formulations of anti-PCSK9 siRNA (ALN-PCS) or subcutaneous inclisiran (27–29). In these phase 1 and 2 trials, plasma PCSK9 and LDL-C levels were evaluated for 4 weeks (for ALN-PCS) or up to 8 months (for inclisiran) of treatment with single and multiple doses of the siRNA compounds, with doses varying from 0.015 to 0.4 mg/kg for ALN-PCS and from 25 to 800 mg for inclisiran. Only a small portion of healthy subjects (13%) received concomitant treatment with statins, while all hypercholesterolemia subjects received statins as a standard of care. To independently evaluate and compare the plasma LDL-C response to PCSK9 lowering under treatment with mAb- or siRNA-based modalities, parameters of PCSK9 effects on LDL-R turnover were fitted separately: for mAbs (alirocumab and evolocumab) and for siRNA (inclisiran and ALN-PCS).

In a third step, the model was calibrated by estimating plasma PK parameters of RG-7652 as well as HDL-C and Lp(a) responses to alirocumab and RG-7652 treatment (30). In the RG-7652 phase 1 study, healthy subjects were assigned to receive a range of single doses from 10 to 800 mg or two doses (on days 1 and 14) from 40 to 150 mg, with PK and Lp(a) measurements being evaluated for up to 16 weeks. Plasma HDL-C and Lp(a) responses over time were available in two phase 1 clinical trials of alirocumab and were used in step 1 of this model-development workflow.

In a fourth step, the model was validated with previously unused PCSK9 and LDL-C aggregated data from an RG-7652 phase 1 trial and an inclisiran phase 2 trial in patients with elevated LDL-C levels and background statin treatment (29, 30). In the inclisiran study, patients were randomly assigned to receive a single dose of placebo or 200, 300, or 500 mg inclisiran or two doses (on days 1 and 90) of placebo or 100, 200, or 300 mg inclisiran. The RG-7652 trial was the same as the one described under the third step.

In a fifth step, data on non-HDL-C, TC, TG, and apoB dynamics from clinical trials described in steps 1 and 2 were used for further model validation. To evaluate responses of apoB, TC, TG, HDL-C, and Lp(a) versus decreases in plasma LDL-C concentrations, week 12 and 24 peak values of the respective biomarkers were compared from available alirocumab and evolocumab data, while, for inclisiran, measurements at the end of weeks 12 and 18 were used.

A detailed description of the available data is given in supplemental Table S1.

### Structure of the mathematical model

The lipoprotein homeostasis model consists of a system of 17 ordinary differential equations. Because only plasma measurements from clinical data were considered in the process of model development and evaluation, no auxiliary compartment other than the plasma compartment was introduced. Additional compartmentalization of the model would have led to model identifiability challenges; that is, experimental data used for parameter estimations could have been described with equal quality by more than one set of parameter estimates, leading to a loss in model identifiability and in predictive power.

Plasma PCSK9 levels were represented by a turnover rate according to equation 1. Because under steady-state conditions system variables do not change over time, and corresponding values can thus be fixed according to respective baseline levels, the synthesis rate in a turnover equation can be explicitly expressed through the metabolite baseline level multiplied by an elimination constant. This allows one to account for the broad variability in baseline values typically encountered in different patient populations and study arms for plasma PCSK9, LDL-C, and Lp(a) turnovers (equations 1, 2, and 4, respectively). The PCSK9 variable was introduced in nmol units to capture PCSK9 binding with anti-PCSK9 mAbs, represented in the model by alirocumab, evolocumab, and RG-7652, assuming a 1:1 molecular binding ratio. PCSK9 plasma concentrations were subsequently calculated by dividing the derived PCSK9 quantities by plasma volume (2.75 l).

(Eq. 1) $$\frac{\mathrm{dPCSK}9}{\mathrm{dt}}\left[\mathrm{nmol}\right]=k_{\mathrm{PCSK}9_{\deg}}\times {\mathrm{Baseline}}_{\mathrm{PCKS}9}-k_{\mathrm{PCSK}9_{\deg}}\times \mathrm{PCSK9}$$

where $k_{\mathrm{PCSK}9_{\deg}}$ is the PCSK9 elimination constant and *Baseline_PCKS9_* is the baseline value of PCSK9 taken directly from each arm of each trial.

Because the low-density fraction of lipoproteins is formed as a result of VLDL hydrolysis of TGs by lipoprotein lipases, we implemented a direct linear relationship between the production rate of LDL-C and VLDL-C, while LDL-C clearance from plasma is regulated by the relative changes in absolute LDL-R quantity that represent the availability of receptors relative to the baseline condition (equation 3).

(Eq. 2) $$\begin{array}{c} \frac{\mathrm{dLDLc}}{\mathrm{dt}}[\mathrm{mg}/\mathrm{dL}]=k_{{\mathrm{LDLc}}_{\deg}}\times {\mathrm{Baseline}}_{\mathrm{LDLc}}\times \left(\frac{\mathrm{VLDLc}}{{\mathrm{Baseline}}_{\mathrm{VLDLc}}}\right)\\ -k_{{\mathrm{LDLc}}_{\deg}}\times \mathrm{LDLc}\times \mathrm{LDLr} \end{array}$$

In equation 2, $k_{{\mathrm{LDLc}}_{\deg}}$represents the elimination constant of LDL-C; *Baseline_LDLc_* and *Baseline_VLDLc_* are baseline values of LDL-C and VLDL-C, respectively; and *LDLr* is the ratio of LDL-R numbers relative to baseline, as described in equation 3. It should be noted that the model does not explicitly take into account time-dependent changes in VLDL-C levels due to *i*) a lack of significant VLDL-C changes under treatment with anti-PCSK9 mAbs or siRNA and *ii*) difficulties in performing simultaneous parametrization of VLDL-C clearance and transformation to LDL-C. LDL-R turnover was described by an apparent degradation constant, with PCSK9-mediated elimination. The elevation in PCSK9 levels above baseline drives the degradation of LDL-Rs, which increases LDL-C concentration in the systemic circulation. Conversely, by removing PCSK9 from plasma, the ratio of free LDL-Rs increased, further contributing to the clearance of LDL-C. In order to account for the endocytic internalization of LDL-Rs, a simplified LDL-C influence mechanism was introduced in a similar fashion to account for the increase in free LDL-Rs in response to decreased amounts of LDL particles in plasma.

(Eq. 3) $$\begin{array}{c} \frac{\mathrm{dLDLr}}{\mathrm{dt}}\left[-\right]=k_{{\mathrm{LDLr}}_{\mathrm{turn}}}-k_{{\mathrm{LDLr}}_{\mathrm{turn}}}\times \mathrm{LDLr}\\ \times {\left(\frac{\mathrm{PCSK}9}{{\mathrm{Baseline}}_{\mathrm{PCSK}9}}\right)}^{n1}\times {\left(\frac{\mathrm{LDLc}}{{\mathrm{Baseline}}_{\mathrm{LDLc}}}\right)}^{n2} \end{array}$$

Here, *LDLr(t)* represents the ratio of free LDL-Rs; $k_{{\mathrm{LDLr}}_{\mathrm{turn}}}$ is the LDL-R turnover coefficient describing the apparent half-life of the receptor; coefficient *n*1 is introduced to account for the nonlinearity between plasma PCSK9 decrease and plasma LDL-C response and is estimated using plasma LDL-C concentrations; and *n*2 quantifies the negative feedback between LDL-C concentration changes relative to baseline and amount of LDL-Rs.

The fourth turnover rate in the model represents the dynamics of Lp(a), an LDL-like particle containing apolipoprotein (a). According to the current view of Lp(a) catabolism, Lp(a) metabolism is considered to be mediated, at least partially, by LDL-Rs (31). This was implemented in the model by introducing two elimination constants representing LDL-R-mediated and non-LDL-R-mediated clearances of Lp(a).

(Eq. 4) $$\begin{array}{c} \frac{\mathrm{dLpA}}{\mathrm{dt}}\left[\mathrm{mg}/\mathrm{dL}\right]=\left(k_{{\mathrm{LpA}}_{\deg}}+k_{{\mathrm{LpA}}_{\deg2}}\right)\times {\mathrm{Baseline}}_{\mathrm{LpA}}\\ -\left(k_{{\mathrm{LpA}}_{\deg}}+k_{{\mathrm{LpA}}_{\deg2}}\times \mathrm{LDLr}\right)\times \mathrm{LpA} \end{array}$$

where $k_{{\mathrm{LpA}}_{\deg}}$ and $k_{{\mathrm{LpA}}_{\deg2}}$ are degradation constants for, respectively, non-LDL-R-mediated and LDL-R-mediated clearances of Lp(a), and *Baseline_LpA_* is the baseline Lp(a) level.

The overall plasma TG pool in the fasting state is primarily divided between VLDL (78%) and LDL (22%) particles (32, 33) and was implemented in equation 5 as follows:

(Eq. 5) $$\mathrm{TG}\left[\%\right]=\left(\frac{\mathrm{VLDLc}}{{\mathrm{Baseline}}_{\mathrm{VLDLc}}}\right)\times \lambda_{\mathrm{tgVLDL}}+\left(\frac{\mathrm{LDLc}}{{\mathrm{Baseline}}_{\mathrm{LDLc}}}\right)\times \lambda_{\mathrm{tgLDL}}$$

*TG* reflects the change from baseline in plasma TG concentration and follows VLDL-C and LDL-C dynamics, and $\lambda_{\mathrm{tgVLDL}}$ and $\lambda_{\mathrm{tgLDL}}$ are the proportions of total plasma TGs in VLDL and LDL at baseline, respectively, under the assumption of a stable cholesterol-to-TG ratio in the respective particles.

In addition, we introduced HDL-C as a model parameter, inversely related to plasma TGs, that captures the action of cholesteryl ester transfer protein (CETP) (34) and allowed us to account for total plasma cholesterol and non-HDL cholesterol in the model:

(Eq. 6) $$\mathrm{HDLc}[\mathrm{mg}/\mathrm{dL}]=\mathrm{HDL}c_{\mathrm{bl}}\times \left(1-\lambda_{\mathrm{tg}}\times \frac{\mathrm{LDLc}}{{\mathrm{Baseline}}_{\mathrm{LDLc}}}\right)$$

(Eq. 7) TC[mg/dL]=HDLc+LDLc+VLDLc

(Eq. 8) NonHDLc[mg/dL]=TC−HDLc

Combining equations 7 and 8 results in non-HDL-C being expressed as the sum of plasma LDL-C and VLDL-C; these equations, taken together, represent the apoB-containing lipoprotein fraction in plasma. Thus, we established a linear relationship between non-HDL-C and the apoB biomarker (equation 9) using a coefficient calculated by a cross-comparison of baseline non-HDL-C and apoB values from every study used in the development of the model. To account for differences in TC-to-apoB ratios between VLDL versus LDL particles, the apoB percentage change from baseline was expressed through LDL-C and VLDL-C changes from baseline on the basis of available data and clinical evidence that LDL contributes approximately 90% to plasma apoB content in healthy subjects (35):

(Eq. 9) $$\begin{array}{c} \mathrm{ApoB}\left[\mathrm{mg}/\mathrm{dL}\right]=\lambda_{\mathrm{apoB}}\times \left({\mathrm{Baseline}}_{\mathrm{LDLc}}+{\mathrm{Baseline}}_{\mathrm{VLDLc}}\right)\\ \times \left(\lambda_{{\mathrm{ApoB}}_{\mathrm{LDLc}}}*\frac{\mathrm{LDLc}}{{\mathrm{Baseline}}_{\mathrm{LDLc}}}+\lambda_{{\mathrm{ApoB}}_{\mathrm{VLDLc}}}\times \frac{\mathrm{VLDLc}}{{\mathrm{Baseline}}_{\mathrm{VLDLc}}}\right) \end{array}$$

Overall, the model successfully captured key features of lipoprotein homeostasis in plasma in minimalistic yet mechanistic terms.

To incorporate the effects of anti-PCSK9 antibodies (mAbs) on cholesterol metabolism, one-compartment PK models with first-order absorption, linear elimination, and no delays were used to describe the plasma PK profiles of subcutaneous alirocumab, evolocumab, and RG-7652 mAbs, with incorporation of target-mediated drug disposition. The amounts of free antibody and free PCSK9 in plasma were both derived in nmol units by recalculating the anti-PCSK9 compound dose from mg to nmol using equation 10:

(Eq. 10) $${\mathrm{dose}}_{\mathrm{nmol}}=10^{6}\times \frac{{\mathrm{dose}}_{\mathrm{mg}}}{{\mathrm{Molecular weight}}_{g/\mathrm{mol}}}$$

This allowed us to implement binding kinetics between plasma mAbs and plasma PCSK9 directly into differential equations of the PK models, as well as PCSK9 turnover, based on the assumption that the PCSK9-to-mAb binding ratio is always 1:1 (36), as reflected in equations 11–19:

(Eq. 11) $$\frac{{\mathrm{dAd}}_{\mathrm{aliro}}}{\mathrm{dt}}[\mathrm{nmol}]=-k_{{\mathrm{abs}}_{\mathrm{aliro}}}\times {\mathrm{Ad}}_{\mathrm{aliro}}$$

(Eq. 12) $$\begin{array}{c} \frac{{\mathrm{dAc}}_{\mathrm{aliro}}}{\mathrm{dt}}\left[\mathrm{nmol}\right]=k_{{\mathrm{abs}}_{\mathrm{aliro}}}\times Ad_{\mathrm{aliro}}-{\mathrm{CL}}_{\mathrm{aliro}}\times \frac{{\mathrm{Ac}}_{\mathrm{aliro}}}{{\mathrm{Vd}}_{\mathrm{aliro}}}-{\mathrm{kon}}_{\mathrm{aliro}}\\ \times \frac{{\mathrm{Ac}}_{\mathrm{aliro}}}{{\mathrm{Vd}}_{\mathrm{aliro}}}\times \mathrm{PCSK}9+{\mathrm{kon}}_{\mathrm{aliro}}\times {\mathrm{Kd}}_{\mathrm{aliro}}\times \mathrm{comA} \end{array}$$

(Eq. 13) $$\begin{array}{c} \frac{\mathrm{dcomA}}{\mathrm{dt}}\left[\mathrm{nmol}\right]={\mathrm{kon}}_{\mathrm{aliro}}\times \frac{{\mathrm{Ac}}_{\mathrm{aliro}}}{{\mathrm{Vd}}_{\mathrm{aliro}}}\times \mathrm{PCSK}9-{\mathrm{kon}}_{\mathrm{aliro}}\\ \times {\mathrm{Kd}}_{\mathrm{aliro}}\times \mathrm{comA}-\frac{{\mathrm{CL}}_{\mathrm{aliro}}}{{\mathrm{Vd}}_{\mathrm{aliro}}}\times \mathrm{comA} \end{array}$$

(Eq. 14) $$\frac{{\mathrm{dAd}}_{\mathrm{evolo}}}{\mathrm{dt}}\left[\mathrm{nmol}\right]=-k_{{\mathrm{abs}}_{\mathrm{evolo}}}\times {\mathrm{Ad}}_{\mathrm{evolo}}$$

(Eq. 15) $$\begin{array}{c} \frac{{\mathrm{dAc}}_{\mathrm{evolo}}}{\mathrm{dt}}\left[\mathrm{nmol}\right]=k_{{\mathrm{abs}}_{\mathrm{evolo}}}\times {\mathrm{Ad}}_{\mathrm{evolo}}-{\mathrm{CL}}_{\mathrm{evolo}}\times \frac{{\mathrm{Ac}}_{\mathrm{evolo}}}{{\mathrm{Vd}}_{\mathrm{evolo}}}-{\mathrm{kon}}_{\mathrm{evolo}}\\ \times \frac{{\mathrm{Ac}}_{\mathrm{evolo}}}{{\mathrm{Vd}}_{\mathrm{evolo}}}\times \mathrm{PCSK}9+{\mathrm{kon}}_{\mathrm{evolo}}\times {\mathrm{Kd}}_{\mathrm{evolo}}\times \mathrm{comE} \end{array}$$

(Eq. 16) $$\begin{array}{c} \frac{\mathrm{dcomE}}{\mathrm{dt}}\left[nmol\right]+{\mathrm{kon}}_{\mathrm{evolo}}\times \frac{{\mathrm{Ac}}_{\mathrm{evolvo}}}{{\mathrm{Vd}}_{\mathrm{evolvo}}}\times PCSK9-{\mathrm{kon}}_{\mathrm{evolvo}}\\ \times {\mathrm{Kd}}_{\mathrm{evolvo}}\times \mathrm{comE}-\frac{{\mathrm{CL}}_{\mathrm{evolvo}}}{{\mathrm{Vd}}_{\mathrm{evolvo}}}\times comE \end{array}$$

(Eq. 17) $$\frac{{\mathrm{dAd}}_{\mathrm{rg}}}{\mathrm{dt}}\left[\mathrm{nmol}\right]=-k_{{\mathrm{abs}}_{\mathrm{rg}}}\times {\mathrm{Ad}}_{\mathrm{rg}}$$

(Eq. 18) $$\begin{array}{c} \frac{{\mathrm{dAc}}_{\mathrm{rg}}}{\mathrm{dt}}\left[\mathrm{nmol}\right]=k_{{\mathrm{abs}}_{\mathrm{rg}}}\times {\mathrm{Ad}}_{\mathrm{rg}}-{\mathrm{CL}}_{\mathrm{rg}}\times \frac{{\mathrm{Ac}}_{\mathrm{rg}}}{{\mathrm{Vd}}_{\mathrm{rg}}}-{\mathrm{kon}}_{\mathrm{rg}}\\ \times \frac{{\mathrm{Ac}}_{\mathrm{rg}}}{{\mathrm{Vd}}_{\mathrm{rg}}}\times PCSK9+{\mathrm{kon}}_{\mathrm{rg}}\times {\mathrm{Kd}}_{\mathrm{rg}}\times comRG \end{array}$$

(Eq. 19) $$\begin{array}{c} \frac{dcomRG}{dt}\left[nmol\right]={\mathrm{kon}}_{\mathrm{rg}}\times \frac{{\mathrm{Ac}}_{\mathrm{rg}}}{{\mathrm{Vd}}_{\mathrm{rg}}}\times \mathrm{PCSK}9-{\mathrm{kon}}_{\mathrm{rg}}\\ \times {\mathrm{Kd}}_{\mathrm{rg}}\times comRG-\frac{{\mathrm{CL}}_{\mathrm{rg}}}{{\mathrm{Vd}}_{\mathrm{rg}}}\times \mathrm{comRG} \end{array}$$

Here, *Ad_rug_* is the amount of drug in the administration compartment; *Ac_drug_* is the amount of drug in circulation; $k_{{\mathrm{abs}}_{\mathrm{drug}}}$ is a reabsorption rate; *Vd_drug_* is a volume of distribution; *CL_drug_* is a rate of clearance; and *comA(t)*, *comE(t)*, and *comRG(t)* reflect the number of complexes formed between PCSK9 and one of the three anti-PCSK9 mAbs. Corresponding binding kinetics for alirocumab, evolocumab, and RG-7652 were implemented through binding constant *kon* and dissociation constant *Kd*.

To take into account anti-PCSK9 antibody effects, equation 1 describing plasma PCSK9 turnover was modified to yield equation 20:

(Eq. 20) $$\begin{array}{c} \frac{dPCSK9}{dt}\left[nmol\right]=k_{\mathrm{PCSK}9_{\deg}}\times {\mathrm{Baseline}}_{\mathrm{PCSK}9}-k_{PCSK9_{deg}}\\ \times PCSK9-{\mathrm{kon}}_{aliro}\times \frac{{\mathrm{Ac}}_{aliro}}{{\mathrm{Vd}}_{aliro}}\times PCSK9\\ +{\mathrm{kon}}_{aliro}\times {\mathrm{Kd}}_{aliro}\times comA-{\mathrm{kon}}_{evolo}\\ \times {\mathrm{Kd}}_{evolo}\times comE-\frac{{\mathrm{CL}}_{evolo}}{{\mathrm{Vd}}_{evolo}}\times comE\\ -{\mathrm{kon}}_{\mathrm{rg}}\times \frac{{\mathrm{Ac}}_{\mathrm{rg}}}{{\mathrm{Vd}}_{\mathrm{rg}}}\times PCSK9+{\mathrm{kon}}_{\mathrm{rg}}\times {\mathrm{Kd}}_{\mathrm{rg}}\times comRG \end{array}$$

Because information on plasma distribution and liver accumulation of anti-PCSK9 siRNA compounds is rather limited, we considered one-compartment PK models with first-order absorption and linear elimination to describe the effect of inclisiran and ALN-PCS on plasma PCSK9. This may be interpreted as a lumped representation of siRNA amounts in the liver after subcutaneous or intravenous administration of inclisiran or ALN-PCS. The doses of both compounds, as well as their respective dynamics, were introduced in mg (equations 21–24):

(Eq. 21) $$\frac{{\mathrm{dAd}}_{inc}}{dt}\left[mg\right]=-k_{{\mathrm{abs}}_{inc}}\times {\mathrm{Ad}}_{inc}$$

(Eq. 22) $$\frac{{\mathrm{dAc}}_{inc}}{dt}\left[mg\right]=k_{{\mathrm{abs}}_{inc}}\times {\mathrm{Ad}}_{inc}-k_{{\mathrm{el}}_{inc}}\times {\mathrm{Ac}}_{inc}$$

(Eq. 23) $$\frac{{\mathrm{dAd}}_{aln}}{dt}\left[mg\right]=-k_{{\mathrm{abs}}_{aln}}\times {\mathrm{Ad}}_{aln}$$

(Eq. 24) $$\frac{d{\mathrm{Ac}}_{inc}}{dt}[mg]=k_{{\mathrm{abs}}_{aln}}\times {\mathrm{Ad}}_{aln}-k_{{\mathrm{el}}_{aln}}\times {\mathrm{Ac}}_{aln}$$

Here, *Ad_drug_* is the amount of drug in the administration compartment; *Ac_drug_* is the amount of drug in the liver; $k_{{\mathrm{abs}}_{drug}}$ is a liver reabsorption rate; and *k_el drug_* is an elimination constant.

siRNA in hepatocytes directly affects the translation of PCSK9 protein from mRNA, which was implemented in the PCSK9 synthesis rate. To take into account anti-PCSK9 siRNA effects, equation 20 was modified to yield equation 25:

(Eq. 25) $$\begin{array}{c} \frac{dPCSK9}{dt}\left[nmol\right]=k_{PCSK9_{deg}}\times {\mathrm{Baseline}}_{PCSK9}\\ \times \left(1-\frac{{\mathrm{Imax}}_{aln}\times {\mathrm{Ac}}_{aln}}{{\mathrm{Ac}}_{aln}+ID50_{aln}}\right)\times \left(1-\frac{{\mathrm{Imax}}_{inc}\times {\mathrm{Ac}}_{inc}}{{\mathrm{Ac}}_{inc}+ID50_{inc}}\right)\\ -k_{PCSK9_{deg}}\times PCSK9-{\mathrm{kon}}_{aliro}\times \frac{{\mathrm{Ac}}_{aliro}}{{\mathrm{Vd}}_{aliro}}\times PCSK9\\ +{\mathrm{kon}}_{aliro}\times {\mathrm{Kd}}_{aliro}\times comA-{\mathrm{kon}}_{evolo}\\ \times {\mathrm{Kd}}_{evolo}\times comE-\frac{{\mathrm{CL}}_{evolo}}{{\mathrm{Vd}}_{evolo}}\times comE-{\mathrm{kon}}_{\mathrm{rg}}\times \frac{{\mathrm{Ac}}_{\mathrm{rg}}}{{\mathrm{Vd}}_{\mathrm{rg}}}\\ \times PCSK9+{\mathrm{kon}}_{\mathrm{rg}}\times {\mathrm{Kd}}_{\mathrm{rg}}\times comRG \end{array}$$

where the *Imax* and *ID50* and coefficients represent inhibitory properties of siRNA-based therapies on PCSK9 mRNA translation.

### Model parameters

A total of 51 parameters were used (supplemental Table S2), of which 21 were estimated using values from the literature. A fixed-effects modeling procedure was used to estimate the remaining 30 parameters using study-level aggregated data.

An initial set of parameters was chosen for model calibration on the basis of physiological limits available from literature sources. Based on these limits, five sets of physiologically plausible initial values were randomly generated, and parameter estimation was performed for each set of initial values. Estimated parameter values did not depend significantly on initial values, which also supported model identifiability.

Baseline values of PCSK9, LDL-C, HDL-C, and Lp(a), as well as doses and regimens, were set according to published trial-level data for each dosing arm of each study. Missing baseline PCSK9 values in five trials of alirocumab (14, 16, 18–20) were imputed using a median PCSK9 value at baseline calculated across the rest of the alirocumab trials. Information on baseline VLDL-C levels was limited to clinical trials of evolocumab. For the remaining studies of other anti-PCSK9 compounds, the baseline VLDL-C level was taken as the median value calculated across all available baseline VLDL-C data from evolocumab trials (23–26).

### Software

Model development and analyses were performed using the IQR systems pharmacology and pharmacometrics toolbox version 0.8.0 (IntiQuan, Basel, Switzerland) on the basis of R software version 3.4.2. Visualization of model simulations was performed using ggplot2 2.1.0 packages.

## RESULTS

### Mathematical modeling of lipoprotein homeostasis

We developed a QSP model to include key elements of lipoprotein homeostasis, PCSK9 dynamics, as well as features of dose-exposure-target modulation to reproduce experimental data of plasma cholesterol response upon administration of anti-PCSK9 mAb or siRNA compounds (Fig. 1A, supplemental Table S1). Ordinary differential equations were implemented to describe and simulate treatment effects of alirocumab, evolocumab, RG-7652, inclisiran, and ALN-PCS on multiple aspects of cholesterol homeostasis in healthy and hypercholesterolemia subjects. To describe endogenous and reverse cholesterol metabolism pathways within the model, we incorporated essential features such as the cholesterol fraction in LDL particles and their VLDL precursors, LDL-R-mediated clearances of LDL-C and Lp(a), HDL-C turnover, PCSK9 expression influenced by siRNA, and plasma PCSK9 binding by mAbs. Figure 1A schematically depicts all structural elements of the model.

VLDL cleavage by lipoprotein lipases results in the formation of LDL particles (step 1). Their abundance is primarily regulated by LDL-R-mediated clearance (step 2), which subsequently facilitates Lp(a) clearance (step 3). The amount of LDL-Rs exposed on hepatocytes is regulated by the PCSK9 enzyme (step 4). The accumulation of the enzyme results in impaired removal of LDL particles from plasma. The PKs and pharmacodynamics of anti-PCSK9 mAbs and siRNA were also characterized in the model. The administration of siRNA affects PCSK9 turnover by disrupting protein synthesis (step 5), while mAbs bind free PCSK9 in plasma with the subsequent elimination of newly formed complexes (step 6). Elevated levels of TGs also affect the amount of cholesterol within HDL particles, which reflects the action of CETP (step 7). The model describes fasting conditions, whereby non-HDL-C is represented by the sum of cholesterol fractions in LDL and VLDL particles (both of which contain apoB molecules). This, in turn, allows apoB quantities to be expressed as a fraction of non-HDL-C and relative to LDL-C and VLDL-C abundancies, while the sum of non-HDL-C and HDL-C reflects the concentration of TC in plasma. Because TGs are represented mainly through VLDL and LDL particles, changes in TG levels are linked to cholesterol fractions within corresponding particles.

A total of 51 parameters were used (supplemental Table S2), of which 21 were estimated using values from the literature. The remaining 30 parameters were calibrated on the basis of plasma PCSK9, LDL-C, HDL-C, and Lp(a) study-level data in healthy and hypercholesterolemia subjects using a nonlinear fixed-effects modeling technique. Point-wise finite sample confidence intervals for the estimated parameters were determined via the Fisher information matrix and likelihood profiling (37). The gradient and the Hessian of the objective function were determined with high numerical precision through the use of symbolically derived parameter sensitivity equations. In order to achieve the best model fitting to various lipoprotein and PCSK9 data, model quality was evaluated using multiple criteria: *i*) change in the objective function value (logarithm of likelihood, Akaike information criterion); *ii*) inspection of diagnostic plots; and *iii*) minimization of the residual error. Further details on the model, including parameters and model diagnostics, are given in the supplemental material.

The predictive power of the QSP model was assessed through an external validation exercise: the model was used in a forward-simulation mode by simulating experimental scenarios and predicting plasma LDL-C data previously unused in the model-development process. The following scenarios were simulated for this purpose, with a post hoc verification against existing data (1): single-ascending dose with RG-7657 in the range of 10 to 800 mg (2) and single (200, 300, 500 mg) and multiple (100, 200, 300 mg) doses with inclisiran based on data from a phase 2 trial (27, 30). The model adequately reproduced all of these additional experimental data (Fig. 1B, C), demonstrating its ability to predict plasma LDL-C responses to anti-PCSK9 mAb and siRNA treatments.

Figure 1B shows that the model adequately predicted plasma LDL-C in an RG-7652 single-dose treatment study in healthy subjects. For higher doses of the anti-PCSK9 antibody, most of the LDL-C data fell within the 95% prediction interval, calculated from the median standard error taken across all mAb studies applied for model development (Fig. 1B). Only the lowest 10 mg RG-7652 dose was underpredicted by the model, which can be explained by the simplistic two-compartment structure of the PK model used.

The model was next used to predict the effects of the siRNA-mediated inhibition of PCSK9 synthesis, upon administration of inclisiran, in hypercholesterolemia subjects on statin treatment (Fig. 1C). Previous healthy subject studies of inclisiran demonstrated that, at ∼80% plasma PCSK9 depletion, plasma LDL-C is reduced by ∼50% (28). The model demonstrates that in hypercholesterolemia subjects, a 50% decrease of LDL-C by inclisiran is achieved with a 10% lesser reduction in PCSK9, which could not be explained by differences in baseline levels alone. The limited amount of data prevents us from drawing specific conclusions; however, we suggest that the underprediction of plasma LDL-C reduction in the phase 2 inclisiran trial may be related to stimulated PCSK9 turnover through SREBP2 activation caused by background statin treatment, as well as the greater contribution of LDL-R-mediated clearance to plasma LDL-C turnover (38).

Overall, this external validation exercise, based on newly simulated scenarios and verified against independent experimental data sets, demonstrates the predictive power of this mechanistic model. It may thus be used to simulate efficacy outcomes of previously untested dosing regimens, including an exploration of the dynamic interplay of the underlying molecular biology and biomarkers in vivo, in response to selected anti-PCSK9 therapies.

### Characteristics of PCSK9 and LDL-C time profiles

**Fig. 2.**
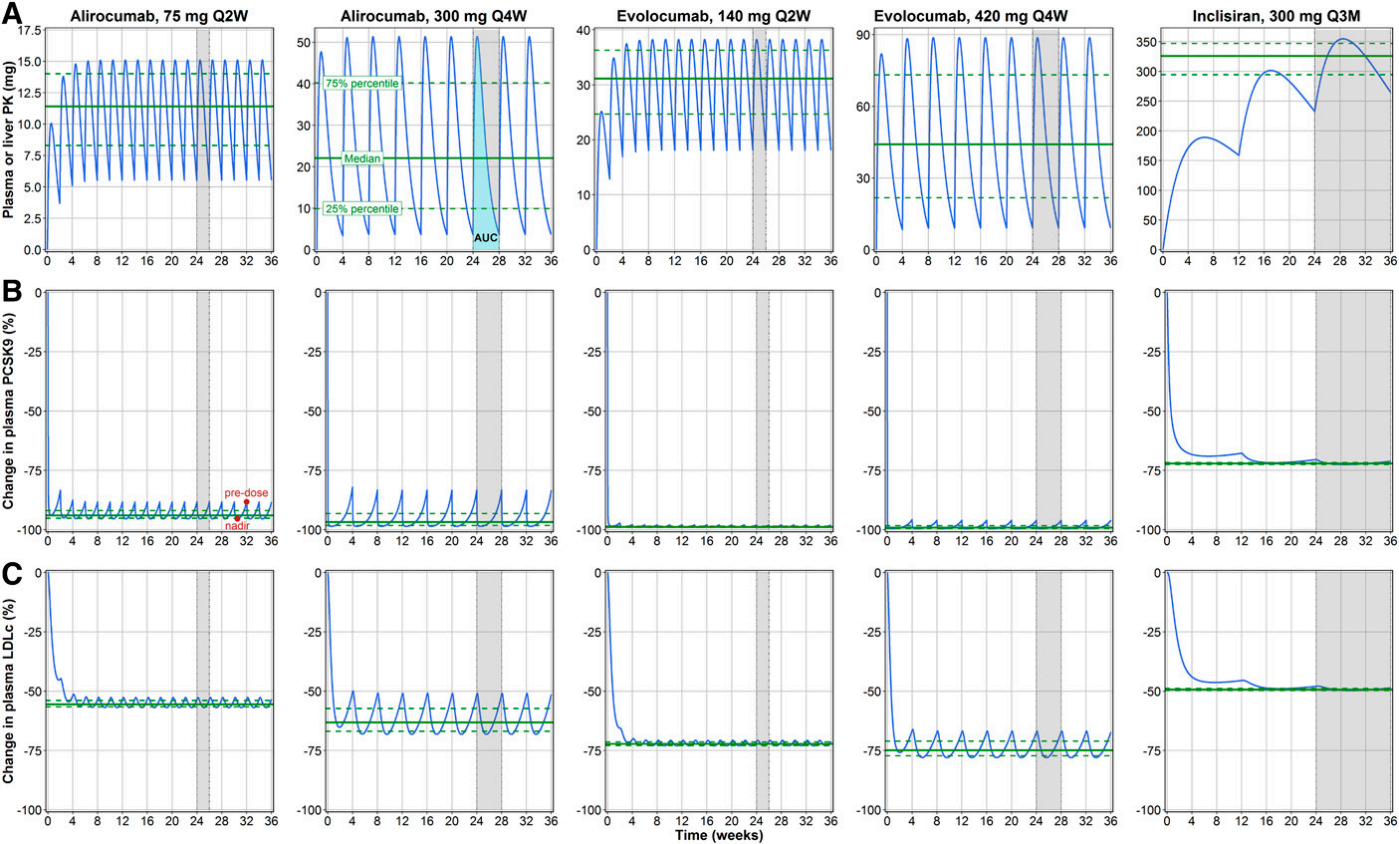
Model-based predictions illustrating the dynamic interplay among PKs (A), key biomarkers, plasma PCSK9 (B), and LDL-C (C). Mean plasma PK, PCSK9, and LDL-C responses to the various compounds and dosing regimens were simulated using the model (blue line) for 36 weeks of consecutive treatment. The median (green solid line) and interquartile range (green dashed line) were calculated for quasi-steady-state periods between doses (shaded gray area).

Detailed longitudinal data reflecting time-dependent changes of molecular signals, as incorporated into the model, allowed us to benchmark anti-PCSK9 mAb and siRNA modalities and to predict time-dependent molecular and biomarker responses under various treatment scenarios, including compound plasma PKs, liver exposure, PCSK9, and LDL-C (**Fig. 2**).

Literature data on mAbs clearly indicate that the profile of plasma free PCSK9 closely follows the PKs of the antibody, indicating a negligible delay in response to treatment; this is reflected in the model by a relatively short apparent half-life of free plasma PCSK9, estimated to be ∼11 h following mAb administration. PK profiles between inclisiran versus mAb modalities, however, differed significantly: once per 4 week administration of 300 mg alirocumab or 420 mg evolocumab doses caused troughs and peaks in plasma PCSK9 (e.g., from −100% to −80% changes vs. baseline); such variations are not observed for the 300 mg dose of inclisiran, which is administered as infrequently as once per 3 months.

These trough-and-peak oscillations under mAb treatment are also reflected in plasma LDL-C profiles, with a ∼20% difference between trough and peak concentrations for Q4W mAb doses vs. ∼5% for inclisiran Q3M administration. The discrepancy between plasma LDL-C and PCSK9 profiles can be explained by differences in the half-lives between plasma PCSK9 and LDL-C, which, for LDL-C, is ∼3 days.

To correctly evaluate the efficacy profile of anti-PCSK9 therapies, it is thus necessary to account for the measurement times of both plasma PCSK9 and LDL-C or use integrative metrics such as AUC or mean plasma concentrations across selected time periods. Model-based simulations (**Table 1**) show that the peak reduction of plasma PCSK9 during a dose interval is ∼25% more effective for mAbs versus inclisiran. However, inclisiran-mediated inhibition of PCSK9 production is more stable over time, resulting in a similar decrease in PCSK9 versus 300 mg Q4W alirocumab based on predose concentrations of the biomarker. Similar effects were observed for plasma LDL-C concentrations as well.

**TABLE 1.** Model-derived statistics of plasma PCSK9 and LDL-C inhibitions (percentage change from baseline) in response to anti-PCSK9 treatment with different compounds and dosing regimens

Biomarker	Drug	Dose (mg)	Predose Concentration	Trough Concentration	Median (Interquartile Range)	AUC (%/day)
PCSK9	Alirocumab	75 Q2W	−88.3	−95.4	−94 (−95.1, −91.9)	−93.3
		300 Q4W	−83.3	−98.6	−96.8 (−98.2, −93.2)	−95.1
	Evolocumab	140 Q2W	−98	−99	−98.8 (−99, −98.5)	−98.7
		420 Q4W	−96	−99.6	−99.2 (−99.5, −98.3)	−98.8
	Inclisiran	300 Q3M	−70.5	−72.6	−72.2 (−72.5, −71.7)	−72
LDL-C	Alirocumab	75 Q2W	−52.7	−56.9	−55.5 (−56.5, −53.9)	−55.2
		300 Q4W	−50.9	−68.2	−63.2 (−66.9, −57.3)	−61.8
	Evolocumab	140 Q2W	−70.7	−73.1	−72.3 (−72.9, −71.4)	−72.1
		420 Q4W	−66.8	−78	−74.9 (−77.2, −71.1)	−73.9
	Inclisiran	300 Q3M	−47.9	−49.7	−49.3 (−49.6, −48.9)	−49.3

### PCSK9-LDL-C relationship

Having elucidated longitudinal responses of PCSK9 and LDL-C for various dosing regimens, we sought to determine potential differences in the plasma PCSK9/LDL-C relationship, under anti-PCSK9 mAb and siRNA treatments, through model-based simulations. A full spectrum of PCSK9 inhibition levels was simulated using two previously identified sets of parameters that reflect plasma PCSK9 impact on LDL-R turnover. To this end, alirocumab, evolocumab, and inclisiran treatments were simulated for 36 weeks under a broad range of treatment regimens (Q2W; Q4W; and continuous intravenous) and doses starting from 1 mg to 10-fold greater than marketed doses. Plasma PCSK9 and LDL-C levels were then compared, at different time points, following the last dosing interval.

**Fig. 3.**
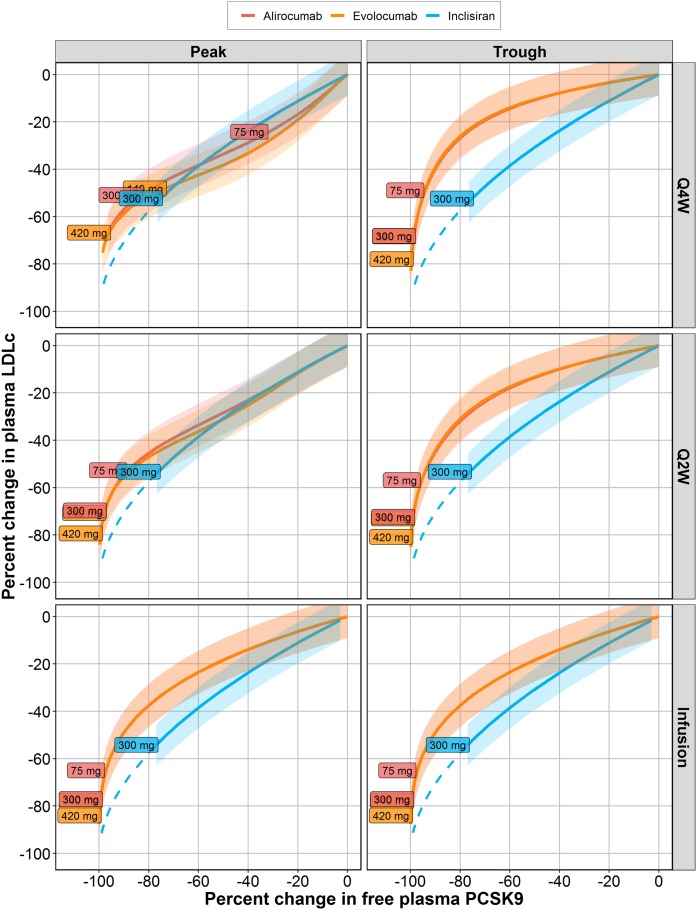
Model simulations using various dosing schedules of alirocumab, evolocumab, and inclisiran. Comparison of plasma PCSK9 and LDL-C levels for the different compounds. Peak and trough concentrations were estimated for weeks 34–36 of a Q2W regimen and for weeks 32–36 of a Q4W regimen. For a continuous infusion, last-point concentrations at week 36 of treatment were analyzed. Colored lines with shaded area represent mean model prediction with a 95% confidence interval. Boxes with numbers reflect the position of the marketed doses (for alirocumab and evolocumab) or phase 3 dose (inclisiran) on the curves. The blue dotted line represents model-based interpolation of plasma PCSK9 reduction for siRNA-based compounds. Values are presented in percentage change from baseline.

As stated previously, regimens and measurement times are important considerations for a correct evaluation of the relationship between plasma PCSK9 and LDL-C, as shown in **Fig. 3**. With respect to peak values, mAb treatments result in a greater reduction in plasma LDL-C versus inclisiran, at 0% to 80% of PCSK9 inhibition levels, while less effective LDL-C reduction for mAbs can be observed when comparing trough concentrations of PCSK9 and LDL-C. To account for compound differences in plasma PKs versus liver exposure, we performed simulations based on continuous intravenous infusion of three different compounds. These simulations show that, at equal levels of plasma PCSK9 lowering, levels of LDL-C reductions were comparable across treatment modalities. They support the hypothesis that, at equal levels of plasma PCSK9 lowering, levels of LDL-C reductions can be comparable across modalities (−30% to −40% plasma LDL-C drop under 60% decrease of PCSK9, with overlapping 95% confidence intervals).

Our model-based simulations of the PCSK9/LDL-C relationship showed that plasma PCSK9 inhibition in response to inclisiran treatment, in contrast to mAbs, reaches saturation at ∼80% PCSK9 reduction from baseline, when a further increase in the dose does not result in a further reduction in plasma PCSK9. This result may be explained by the fact that siRNA-based compounds mainly are taken up by and knock down the PCSK9 production in the liver, while PCSK9 expression in other tissues remains unaffected (39). Hence, the LDL-C-lowering potential of antibodies might be superior to that of siRNA.

To explore the potential of siRNA-based compounds to inhibit LDL-C, we performed simulations with extrapolated thresholds of PCSK9 synthesis inhibition from roughly 80% to 95% change from baseline. Simulation results show that siRNA-mediated PCSK9 inhibition greater than 90% would allow reaching plasma LDL-C inhibition levels comparable to those of mAbs, and exceeding the 95% threshold may result in greater LDL-C reduction versus an mAb treatment.

### Simulations of other biomarkers

**Fig. 4.**
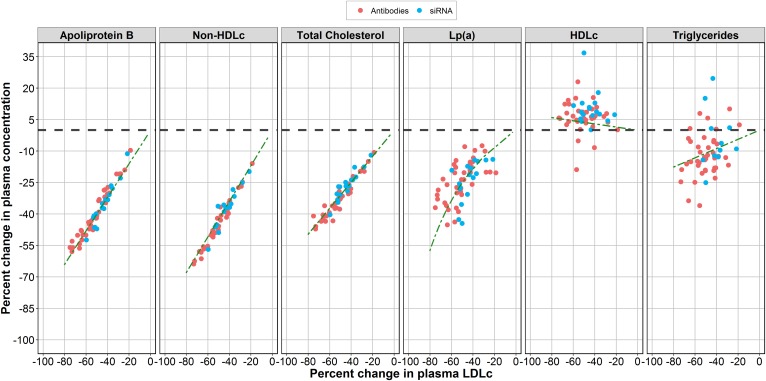
Lipoprotein and lipid biomarker responses following plasma LDL-C lowering under different anti-PCSK9 therapies. Predose values of apoB, non-HDL-C, TC, Lp(a), HDL-C, TGs, and LDL-C from clinical data on alirocumab, evolocumab, RG-7652 (red dots), as well as inclisiran with ALN-PCS (blue dots) were compared with model-generated values (green dashed curves). Values are presented as percentage change from baseline.

The mechanistic model of lipid metabolism, which we developed, incorporates additional lipid biomarkers beyond plasma PCSK9 and LDL-C, including HDL-C, Lp(a), as well as non-HDL-C, TC, apoB, and TGs. We next used the model to determine how these various lipid biomarkers are modulated, dynamically, under siRNA and mAb treatments. We performed an additional validation of the model by comparing model-derived peak values of biomarkers with values from 17 studies of alirocumab, evolocumab, RG-7652, inclisiran, and ALN-PCS (**Fig. 4**). The model adequately captured trends in apoB, non-HDL-C, TC, and Lp(a) dynamics upon treatment-induced decreases in plasma LDL-C and irrespective of treatment modality type. Relationships between plasma LDL-C, HDL-C, and TGs were more ambiguous, as illustrated by experimental data, suggesting that TG metabolism is primarily driven by non-LDL-C-related factors, and, in order to successfully predict the TG/HDL-C index for anti-PCSK9 treatment, additional aspects of lipid metabolism should be incorporated into the model.

Model-based simulations of a variety of lipoprotein and lipid plasma biomarkers revealed no significant differences between mAb- and siRNA-based anti-PCSK9 modalities, which may suggest that siRNA-mediated cleavage of PCSK9 mRNA within hepatocytes would not incur further effects on these markers versus mAbs.

## DISCUSSION

In this work, we developed and validated a quantitative, mechanism-based systems pharmacology model to benchmark lipoprotein-lowering qualities of two classes of anti-PCSK9 pharmacological treatment modalities: mAbs and siRNA. The model was based on 17 clinical trials of alirocumab, evolocumab, RG-7652, inclisiran, and ALN-PCS, with longitudinal measurements of plasma PCSK9, LDL-C, apoB, non-HDL-C, TC, HDL-C, TGs, and Lp(a). The mechanistic structure of lipid metabolism was modeled on the basis of a system of biological and physiological relationships, while minimizing the number of assumptions made and keeping the model fit for predictive purposes. In the model, HDL-C levels were linked primarily to the abundance of TG molecules in plasma, which reflects the action of CETP: a plasma protein that exchanges cholesterol ester from HDL particles for TGs in other lipoprotein particles. Because PCSK9 affected the clearance of LDL particles only, the amount of VLDL particles, and therefore their cholesterol fraction, were not affected by anti-PCSK9 treatment. Hence, the VLDL-C concentration was fixed at baseline level and did not change in response to treatment. Further model developments may be considered, for example, by evaluating the effects of statins. In addition, while the modeled Lp(a) clearance was partially mediated by LDL-Rs, the true mechanisms of Lp(a) catabolism are largely unknown.

By jointly analyzing the dynamics of plasma PCSK9 and LDL-C, we first determined that it is imperative to account for differences in the PK profiles of the compounds to appropriately compare the efficacy between the two treatment modalities (mAbs vs. siRNA). Significant kinetic variations in plasma PCSK9 levels (Q4W dosing regimens of mAbs) were also reflected in plasma LDL-C variations, though to a lesser magnitude, in contrast to a stable, long-term inhibition of both biomarkers under inclisiran treatment. Hence, using predose or peak concentration values might only be misleading if one attempts to estimate overall LDL-C-lowering qualities across treatment modalities. The presence of greater plasma LDL-C variations under monthly mAbs administration compared with inclisiran could differentiate siRNA compounds from mAbs, although the impact of plasma LDL-C changes over time on MACE clinical end points requires further investigation.

A comparison of PCSK9 and LDL-C reductions using integrative metrics such as AUC and model-based simulations under intravenous infusion revealed that the LDL-C-lowering potential is similar between mAb- and siRNA-based compounds. However, intracellular inhibition of PCSK9 translation by siRNA in hepatocytes did not result in the complete elimination of plasma PCSK9. We hypothesized such a result to be primarily related to a combination of two factors: high tissue location specificity of inclisiran and ALN-PCS to the liver and the presence of PCSK9 mRNA in tissues other than the liver. As a result, siRNA might completely block PCSK9 synthesis in the liver, while the 20% remaining PCSK9 in plasma may originate from other tissues. This may point to siRNA-based therapies being potentially less effective than mAbs. However, our model-based predictions demonstrate noninferiority of siRNA to mAbs if a 90% threshold of PCSK9 inhibition were to be met, with a further siRNA-induced decrease in PCSK9, resulting in superior reduction in LDL-C, compared with, for example, a Q4W mAb regimen. Based on simulations with an siRNA, a greater than 95% reduction in plasma PCSK9 would result in superior plasma LDL-C reduction versus current drugs. Taken together, these quantitative simulations may support drug research and development efforts in terms of modality choices and requirements for molecular design and pharmacological properties of novel anti-PSCK9 candidates. However, whether or not this would lead to an improvement in the MACE clinical end point is not known.

Cholesterol metabolism is a complex process with a plethora of interdependent markers. We sought to evaluate how different anti-PCSK9 modalities (mAbs vs. siRNA) may affect different lipoprotein and lipid biomarkers, aside from plasma PCSK9 and LDL-C. Treatment-dependent reductions in integrative biomarkers such as TC, non-HDL-C, and apoB were linearly dependent on LDL-C levels, an expected result because LDL-C is directly or indirectly included in these markers. However, no differences in these biomarkers were observed between mAbs versus siRNA treatment, indicative of no additional benefits, with respect to non-LDL-C biomarkers, to be gained from the intracellular inhibition of PCSK9 translation in the patient segment investigated (patients with hypercholesterolemia driven by a high abundance of LDL particles) and within the considered range of LDL-C reduction.

For TGs and HDL-C, we did not observe a clear dependency versus LDL-C reduction. TGs, like non-HDL-C and TC, are an integrative marker represented in multiple supramolecular components, largely represented by VLDL particles in the fasting state. We assumed a linear relationship between TGs and LDL-C, with the TG fraction in LDL particles calculated as 22% of the total plasma TG pool. The model did not adequately capture the clinical data at hand, suggesting that TG metabolism primarily relies on non-LDL-dependent mechanisms that are not affected by the anti-PCSK9 treatment and, therefore, are out of scope of the current model.

HDL-C is part of the TC pool in plasma; HDL-C increases by 5% to 10% in virtually every anti-PCSK9 trial. Because plasma TG dynamics were shown to be independent of anti-PCSK9 treatment, HDL-C increases under cholesterol-lowering treatment did not appear to be dose-proportional either, given the available clinical data.

Anti-PCSK9 treatment resulted in plasma Lp(a) lowering, which was correlated with a reduction in plasma LDL-C. While both biomarkers are susceptible to LDL-R-mediated clearance, Lp(a) response is more variable compared with LDL-C. To adequately describe the kinetics of these TG, HDL-C, and Lp(a) markers under various anti-PSCK9 treatment modalities, additional clinical data and of mechanistic details would need to be integrated into the model.

Like any mathematical representation of biological processes, the current model features limitations that arise from both the available data and the chosen model structure. The development of a model that adequately and simultaneously describes dynamic responses of various lipoprotein biomarkers to five different anti-PCSK9 therapies requires guided data mining in combination with various domain experts and from all available open sources. Unfortunately, such clinical data are often reported at an aggregated trial level, with mean or median values calculated per dosing arm. This prevents us from applying mixed-effects modeling and evaluating between-subject variability. However, the model structure, as presented here, allows to account for between-study differences in baseline levels for variables of interest, while the uncertainty in model predictions can be evaluated using a fixed-effects modeling methodology. In addition, to provide an adequate balance between model identifiability and a sufficiently accurate representation of the underlying biology, reasonable simplifications to the description of LDL-R distribution needed to be introduced. Parametrization of LDL-R binding with LDL, PCSK9, or both, with subsequent hepatocyte internalization and degradation or reexposure requires, at a minimum, detailed information on the quantity of unbound LDL-Rs on the surface of hepatocytes, as well as the quantities and internalization rates of LDL-R-PCSK9 and LDL-R-PCSK9-LDL complexes. Such data were not available for this modeling study; following the principle of parsimony, the two main feedback mechanisms that were introduced into the LDL-R equation (see equation 3) were sufficient to successfully identify and parameterize PCSK9-mediated degradation and LDL-C-mediated uptake of the receptors. Based on the same principle, ratios of apoB (VLDL and LDL particles) were fixed and calculated in absolute values on the basis of the ratio of apoB to non-HDL-C, an integrative biomarker comprising LDL-C and VLDL-C measurements. While the linear correlation between non-HDL-C and apoB is well-established and corresponds to well-established physiology (40), the apoB response to non-HDL-C lowering might result in additional effector responses depending on the patient population; thus, one should exercise caution when interpreting the present model-based predictions for populations with baseline VLDL-C levels that would significantly differ from those considered in the model development. While the current model is focused on providing quantitative insights into the behavior of lipoprotein biomarkers under anti-PCSK9 treatment in hypercholesterolemia characterized by high LDL-C levels, it remains to be seen if the same effects were to be observed in other forms of hypercholesterolemia.

Despite these limitations, the model adequately describes the quantities and kinetics of plasma PCSK9, LDL-C, apoB, non-HDL-C, and TC and may serve as a quantitative tool for benchmarking anti-PCSK9 treatment modalities such as mAbs and siRNAs.

The model showed that, although siRNA and mAbs differ in their mechanisms of action and pharmacokinetic profiles, at equal levels of plasma PCSK9 lowering, levels of LDL-C reductions were comparable across drug modalities, and no differences were observed in responses of the spectrum of other lipoprotein and lipid plasma biomarkers within this patient population. The potential limitation of the siRNA-based modality lies in the apparent inability of these compounds to virtually eliminate PCSK9 due to either their mode of action or disposition characteristics. Changing the biochemical and delivery properties of synthesis inhibitors may thus be key in reaching a similar or even superior lowering in circulating PCSK9 levels versus those of mAbs, which would subsequently lead to a similar, or greater, reduction in LDL-C.

## Supplementary Material

Supplemental Data
