# Supplementary material for: Comparative quantitative systems pharmacology modeling of anti-PCSK9 therapeutic modalities in hypercholesterolemia
Source: J Lipid Res. 2019 Jul 10;60(9):1610–21. doi: 10.1194/jlr.M092486 (PMC6718444; doi:10.1194/jlr.M092486)
Supplement: Supplemental Data [file supp_60_9_1610__index.html]

Comparative quantitative systems pharmacology modeling of anti-PCSK9 therapeutic modalities in hypercholesterolemia — Comparative quantitative systems pharmacology modeling of anti-PCSK9 therapeutic modalities in hypercholesterolemia — Supplemental Data 

# Comparative quantitative systems pharmacology modeling of anti-PCSK9 therapeutic modalities in hypercholesterolemia

## Supplemental Data

- Supplemental materials (.pdf, 1.1 MB) - A file with supplemental figures and tables.
