# Supplementary material for: Comparative quantitative systems pharmacology modeling of anti-PCSK9 therapeutic modalities in hypercholesterolemia
Source: J Lipid Res. 2019 Jul 10;60(9):1610–21. doi: 10.1194/jlr.M092486 (PMC6718444; doi:10.1194/jlr.M092486)
Supplement: Supplemental Data [file 10.1194_M092486_jlr.M092486-1.pdf]

Supplementary Material

Manuscript:

Comparative quantitative systems pharmacology modeling of anti-PCSK9 therapeutic modalities in hypercholesterolemia

Authors: V. Sokolov[1], G. Helmlinger[2] (PhD), C. Nilsson[3] (PhD), K. Zhudenkov[1] (PhD), S. Skrtic[3,4] (PhD), B. Hamrén[3] (PhD), K. Peskov[1,5] (PhD), E. Hurt-Camejo[6] (PhD), R. Jansson-Löfmark[6] (PhD)

Affiliations:

[1]M&S Decisions, Moscow, Russia.

[2]Clinical Pharmacology & Safety Sciences, R&D BioPharmaceuticals, AstraZeneca, Boston, USA.

[3] Clinical Pharmacology & Safety Sciences, R&D BioPharmaceuticals, AstraZeneca, Gothenburg, Sweden.

[4]Institute of Medicine at Sahlgrenska Academy, University of Gothenburg, Gothenburg, Sweden.

[5]I.M. Sechenov First Moscow State Medical University of the Russian Ministry of Health.

[6]Cardiovascular, Renal and Metabolism, R&D BioPharmaceuticals, AstraZeneca, Gothenburg, Sweden.

Supplemental Figures and Figure Legends

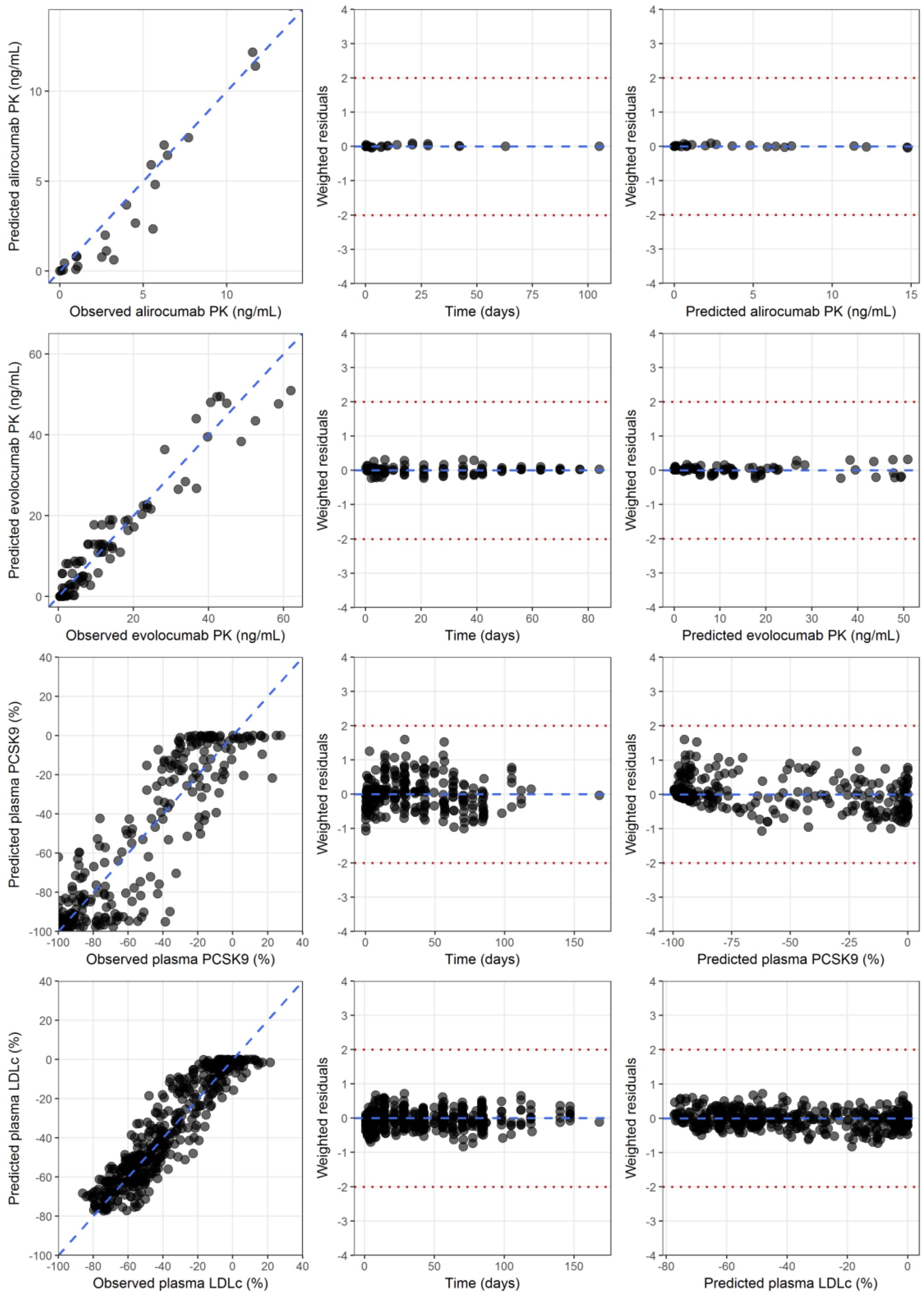

Supplemental Figure S1. Model diagnostics plots for alirocumab plasma PK (A), evolocumab plasma PK (B), free plasma PCSK9 (C) and plasma LDLc (D) under alirocumab or evolocumab treatment. First, second and third columns represent observations *vs*. model predictions, residuals *vs*.

time and residuals *vs*. model predictions, respectively. The straight blue line illustrates full agreement between experimental and calculated values. Experimental data taken from (1–15).

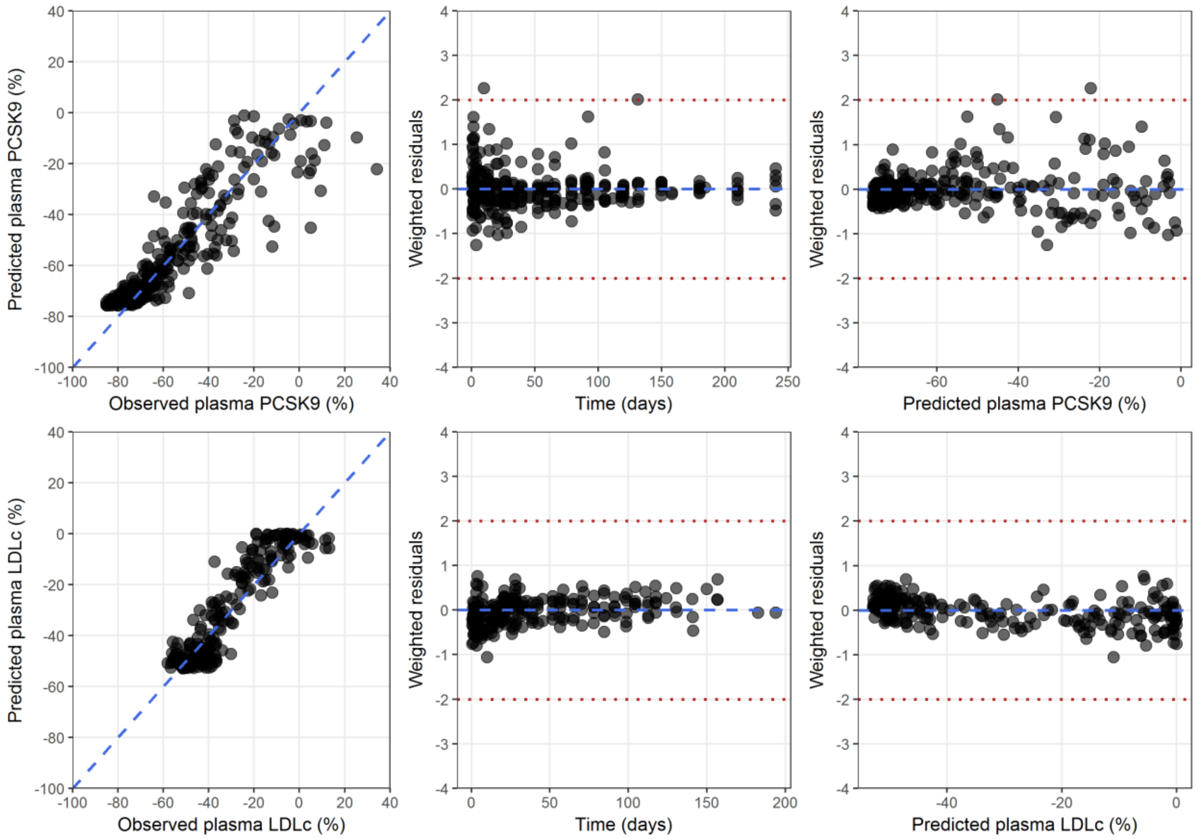

Supplemental Figure S2. Model diagnostics plots for free plasma PCSK9 (A) and plasma LDLc (B) under inclisiran or ALN-PCS treatment. First, second and third columns represent observations *vs*. model predictions, residuals *vs*. time and residuals *vs*. model predictions, respectively. The straight blue line illustrates full agreement between experimental and calculated values. Experimental data taken from (16–18).

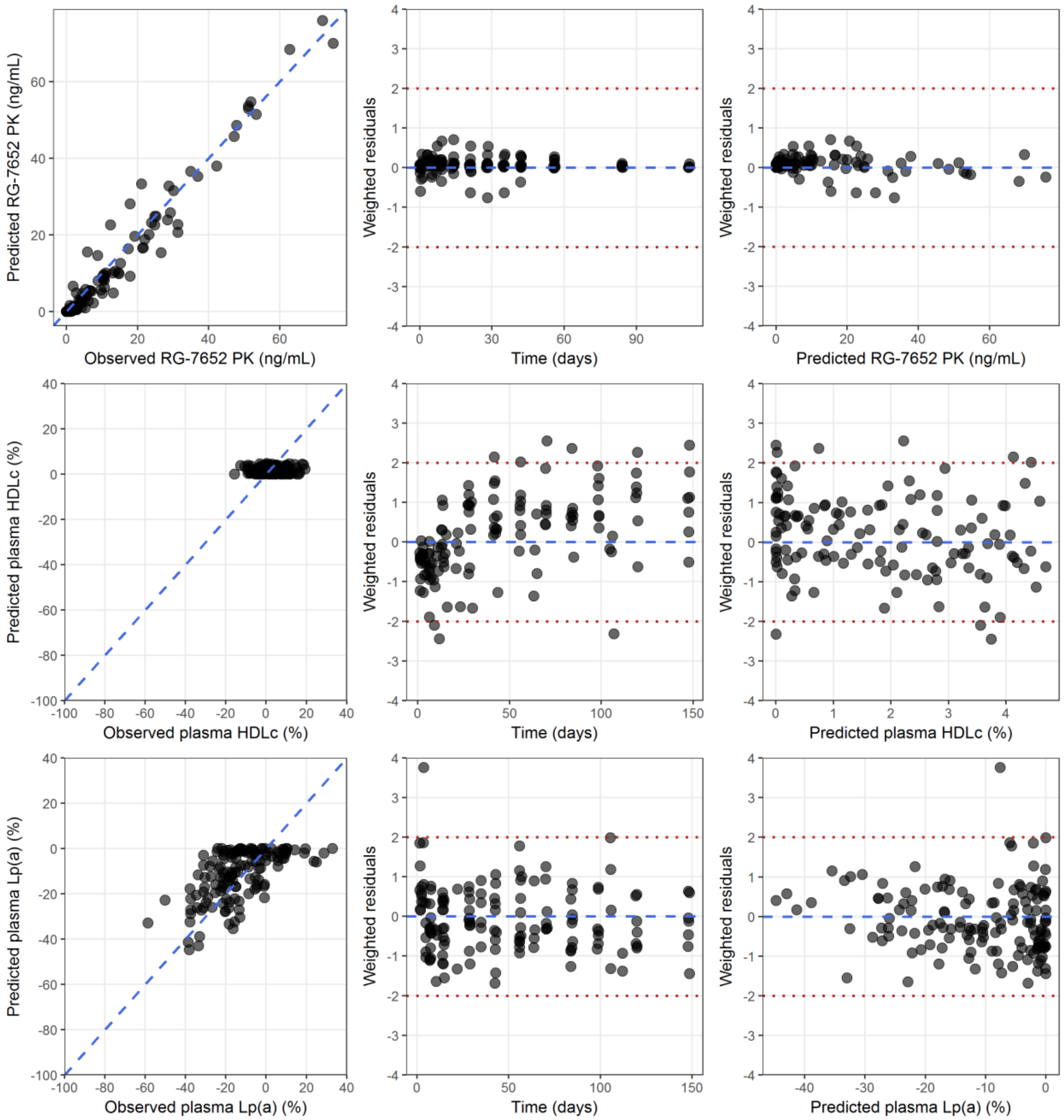

Supplemental Figure S3. Model diagnostics plots for RG-7652 PK (A), plasma HDLc (B) and plasma Lp(a) under treatment with alirocumab, evolocumab, or RG-7652. First, second and third columns represent observations *vs*. model predictions, residuals *vs*. time and residuals *vs*. model predictions respectively. The straight blue line illustrates full agreement between experimental and calculated values. Experimental data taken from (1, 4, 19).

Supplemental Tables and Supporting Information

Supplemental Table S1. Experimental data used for model development

Reference	Clinical trial	Phase	Drug	Available measurements	Treatment	Data points	Population
Lunven et al. 2014 (1)	NCT01785329	1	Alirocumab	PK, PCSK9, LDLc, TC, non-HDLc, HDLc, TG	75 mg, single	23	Healthy, without statins, n=20
Rey et al. 2016 (2)	NCT01723735	1	Alirocumab	PCSK9, LDLc	150 mg, multiple	43	Healthy, with and without statins, n=24
Roth et al. 2014 (3)	NCT01074372	1	Alirocumab	PK, PCSK9	150 mg, single	23	Healthy, without statins, n=10
Stein et al. 2012b (4)	NCT01074372	1	Alirocumab	LDLc, TC, non-HDLc, ApoB, HDLc, Lp(a), PCSK9	50, 100, 150, 250 mg, single	26	Healthy, without statins, n=64
Stein et al. 2012b (4)	NCT01161082	1	Alirocumab	PCSK9, LDLc, TC, non-HDLc, ApoB, HDLc, Lp(a)	50, 100, 150 mg, multiple	35	Healthy, Familial Hypercholesterolemia, with and without statins, n=39
McKenney et al. 2012 (5)	NCT01288443	2	Alirocumab	LDLc	50, 100, 150, 200, 300 mg, multiple	84	Hypercholesterolemia, with statins, n=148
Roth et al. 2012 (6)	NCT01288469	2	Alirocumab	PCSK9, LDLc	150 mg, multiple	65	Hypercholesterolemia, with statins, n=59
Stein et al. 2012a (7)	NCT01266876	2	Alirocumab	LDLc, ApoB	150, 200, 300 mg, multiple	74	Familial Hypercholesterolemia, with statins, n=62
Bays et al. 2015 (8)	NCT01730040 (ODYSSEY OPTIONS I)	3	Alirocumab	LDLc	75 mg, multiple	34	Hypercholesterolemia, with statins, n=104

Cannon et al. 2015 (9)	NCT01644188 (ODYSSEY COMBO II)	3	Alirocumab	LDLc	75 mg, multiple	52	Hypercholesterolemia, with statins, n=467
Stroes et al. 2016 (10)	NCT02023879 (ODYSSEY CHOICE II)	3	Alirocumab	PCSK9, LDLc	75, 150 mg, multiple	65	Hypercholesterolemia, with and without statins, n=129
Fitzgerald et al. 2014 (17)	NCT01437059	1	ALN-PCS	PCSK9, LDLc	0.015, 0.045, 0.090, 0.150, 0.250, 0.400 mg/kg, single	22	Hypercholesterolemia, without statins, n=24
FDA report 20080397	-	1	Evolocumab	PK, PCSK9, LDLc, ApoB	7, 14, 21, 35, 70, 140, 210, 280, 420 mg, single and multiple	64	Healthy, Hypercholesterolemia, with and without statins, n=NA
FDA report 20080398	-	1	Evolocumab	PK, PCSK9, LDLc, ApoB	7, 14, 21, 35, 70, 140, 210, 280, 420 mg, single and multiple	42	Healthy, Hypercholesterolemia, with and without statins, n=NA
Dias et al. 2012 (11)	NCT01133522	1	Evolocumab	PK, PCSK9, LDLc, ApoB	7, 14, 21, 35, 70, 140, 210, 280, 420 mg, single and multiple	54	Healthy, Hypercholesterolemia, with and without statins, n=NA
Giugliano et al. 2012 (12)	NCT01380730 (LAPLACE)	2	Evolocumab	LDLc	70, 105, 140, 280, 350, 420 mg, multiple	65	Hypercholesterolemia, with statins, n=474

Koren et al. 2014 (13)	NCT01375777 (MENDEL)	2	Evolocumab	LDLc	140, 420 mg, multiple	83	Hypercholesterolemia, without statins, n=306
Raal et al. 2012 (14)	NCT01375751 (RUTHER-FORD)	2	Evolocumab	LDLc	350, 420 mg, multiple	12	Familial Hypercholesterolemia, with statins, n=111
Sullivan et al. 2012 (15)	NCT01375764 (GAUSS)	2	Evolocumab	LDLc	280, 350, 420 mg, multiple	43	Hypercholesterolemia, without statins, n=95
Fitzgerald et al. 2017 (16)	NCT02314442	1	Inclisiran	PCSK9, LDLc	25, 100, 125, 250, 300, 500, 800 mg, single and multiple	23	Hypercholesterolemia, with and without statins, n=47
Ray et al. 2017 (18)	NCT02597127 (ORION)	2	Inclisiran	PCSK9, LDLc	100, 200, 300, 500 mg, single and multiple	41	Hypercholesterolemia, with statins, n=309
Baruch et al. 2017 (19)	NCT01609140	1	RG7652	PK, PCSK9, LDLc, ApoB, Lp(a)	10, 40, 150, 300, 600, 800 mg, single and multiple	26	Healthy, Hypercholesterolemia, with and without statins, n=36

Supplemental Table S2. Model parameters

Parameter	Unit	Description	Value	95% confidence interval	Estimation method and References
λ_{apoB}	-	NonHDLc-to-ApoB conversion coefficient	-0.654	-	calculated
V_{pl}	L	Plasma volume	2.75	-	taken from the literature (20)
MW_{PCSK9}	g/mol	PCSK9 molecular weight	-	-	taken from the literature (21)
$Baseline_{PCSK9}$	nmol	Baseline PCSK9	-	-	taken from each arm of each trial
$Baseline_{LDLc}$	mg/dL	Baseline LDLc	-	-	taken from each arm of each trial
$Baseline_{LpA}$	mg/dL	Baseline Lp(a)	-	-	taken from each arm of each trial
$Baseline_{HDLc}$	mg/dL	Baseline HDLc	-	-	taken from each arm of each trial
$Baseline_{VLDLc}$	mg/dL	Median VLDLc level taken from evolocumab and inclisiran studies	23.166	-	taken from each arm of each trial
$k_{PCSK9_{deg}}$	1/day	PCSK9 degradation	1.5	[1.4; 1.61]	fitted
λ_{tg}	-	Influence of TG on HDLc	0.34	[0.23; 0.47]	fitted

$k_{LpA_{deg}}$	1/day	LDLr-independent degradation of Lp(a)	0.09	[0.06; 0.13]	fitted
$k_{LpA_{deg2}}$	1/day	LDLr-dependent degradation of Lp(a)	0.04	[0.03; 0.06]	fitted
$k_{LDLc_{deg}}$	1/day	LDLc clearance	0.231	-	taken from the literature (22)
$k_{LDLr_{turn}}$	1/day	LDLr turnover	3.37	[1.58; 7.21]	fitted
$n2$	-	LDLc influence on LDLr degradation	0.52	[0.44; 0.6]	fitted
**Alirocumab parameters**					
$dose_{aliro}$	mg	Dose	-	-	taken from each arm of each trial
MW_{aliro}	g/mol	Molecular weight	146000	-	taken from the FDA report
$k_{abs_{aliro}}$	1/day	Absorption	0.1	[0.1; 0.11]	fitted
CL_{aliro}	L/day	Clearance	0.52	[0.48; 0.56]	fitted
Vd_{aliro}	L	Volume of distribution	1.37	[1.17; 1.6]	fitted
kon_{aliro}	1/nmol/L	Binding constant	0.94	[0.71; 1.24]	fitted
Kd_{aliro}	nmol/L	Dissociation constant	0.52	[0.38; 0.71]	fitted
$n1$	-	PCSK9 influence on LDLr degradation	0.14	[0.12; 0.17]	fitted
**Evolocumab parameters**					
$dose_{evolo}$	mg	Dose	-	-	taken from each arm of each trial

MW_{evolo}	g/mol	Molecular weight	141800	-	taken from the FDA report
$k_{abs_{evolo}}$	1/day	Absorption	0.095	[0.09; 0.101]	fitted
CL_{evolo}	L/day	Clearance	0.454	[0.433; 0.475]	fitted
Vd_{evolo}	L	Volume of distribution	1.34	[1.233; 1.447]	fitted
kon_{evolo}	1/nmol/L	Binding constant	0.94	-	assumed equal to kon_{aliro}
Kd_{evolo}	nmol/L	Dissociation constant	0.016	-	taken from the FDA report
$n1$	-	PCSK9 influence on LDLr degradation	0.14	[0.12; 0.17]	fitted
**RG-7652 parameters**					
$dose_{rg}$	mg	Dose	-	-	taken from each arm of each trial
MW_{rg}	g/mol	Molecular weight	141800	-	assumed equal to MW_{aliro}
$k_{abs_{rg}}$	1/day	Absorption	0.05	[0.04; 0.05]	fitted
CL_{rg}	L/day	Clearance	0.35	[0.33; 0.37]	fitted
Vd_{rg}	L	Volume of distribution	0.71	[0.61; 0.82]	fitted
kon_{rg}	1/nmol/L	Binding constant	0.94	-	assumed equal to kon_{aliro}

Kd_{rg}	nmol/L	Dissociation constant	0.52	-	assumed equal to Kd_{aliro}
$n1$	-	PCSK9 influence on LDLr degradation	0.14	[0.12; 0.17]	fitted
**Inclisiran parameters**					
$dose_{inc}$	mg	Dose	-	-	taken from each arm of each trial
$k_{abs_{inc}}$	1/day	Absorption	0.04	[0.03; 0.05]	fitted
$k_{el_{inc}}$	1/day	Elimination	0.01	[0.01; 0.02]	fitted
$Imax_{inc}$	-	Maximum PCSK9 inhibition	0.77	[0.75; 0.79]	fitted
$ID50_{inc}$	mg	ID50 of PCSK9 inhibition	21.74	[15.32; 30.85]	fitted
$n1$	-	PCSK9 influence on LDLr degradation	0.26	[0.25; 0.27]	fitted
**ALN-PCS parameters**					
$dose_{aln}$	mg	Dose	-	-	taken from each arm of each trial
$k_{abs_{aln}}$	1/day	Absorption	2.59	[0.7; 9.55]	fitted
$k_{el_{aln}}$	1/day	Elimination	0.13	[0.11; 0.15]	fitted
$Imax_{aln}$	-	Maximum PCSK9 inhibition	0.78	[0.7; 0.84]	fitted
$ID50_{aln}$	mg	ID_{50} of PCSK9 inhibition	2.55	[1.74; 3.75]	fitted

$n1$	-	PCSK9 influence on LDLr degradation	0.26	[0.25; 0.27]	fitted

Supplemental References

1. Lunven, C., T. Paehler, F. Poitiers, A. Brunet, J. Rey, C. Hanotin, and W. J. Sasiela. 2014. A Randomized Study of the Relative Pharmacokinetics, Pharmacodynamics, and Safety of Alirocumab, a Fully Human Monoclonal Antibody to PCSK9, After Single Subcutaneous Administration at Three Different Injection Sites in Healthy Subjects. *Cardiovascular Therapeutics*. **32**: 297–301.

2. Rey, J., F. Poitiers, T. Paehler, A. Brunet, A. T. DiCioccio, C. P. Cannon, H. K. Surks, J. Pinquier, C. Hanotin, and W. J. Sasiela. 2016. Relationship Between Low-Density Lipoprotein Cholesterol, Free Proprotein Convertase Subtilisin/Kexin Type 9, and Alirocumab Levels After Different Lipid-Lowering Strategies. *Journal of the American Heart Association*. **5**: e003323.

3. Roth, E. M., and P. Diller. 2014. Alirocumab for hyperlipidemia: physiology of PCSK9 inhibition, pharmacodynamics and Phase I and II clinical trial results of a PCSK9 monoclonal antibody. *Future Cardiology*. **10**: 183–199.

4. Stein, E. A., S. Mellis, G. D. Yancopoulos, N. Stahl, D. Logan, W. B. Smith, E. Lisbon, M. Gutierrez, C. Webb, R. Wu, Y. Du, T. Kranz, E. Gasparino, and G. D. Swergold. 2012. Effect of a monoclonal antibody to PCSK9 on LDL cholesterol. *N. Engl. J. Med.* **366**: 1108–1118.

5. McKenney, J. M., M. J. Koren, D. J. Kereiakes, C. Hanotin, A.-C. Ferrand, and E. A. Stein. 2012. Safety and Efficacy of a Monoclonal Antibody to Proprotein Convertase Subtilisin/Kexin Type 9 Serine Protease, SAR236553/REGN727, in Patients With Primary Hypercholesterolemia Receiving Ongoing Stable Atorvastatin Therapy. *Journal of the American College of Cardiology*. **59**: 2344–2353.

6. Roth, E. M., J. M. McKenney, C. Hanotin, G. Asset, and E. A. Stein. 2012. Atorvastatin with or without an Antibody to PCSK9 in Primary Hypercholesterolemia. *New England Journal of Medicine*. **367**: 1891–1900.

7. Stein, E. A., D. Gipe, J. Bergeron, D. Gaudet, R. Weiss, R. Dufour, R. Wu, and R. Pordy. 2012. Effect of a monoclonal antibody to PCSK9, REGN727/SAR236553, to reduce low-density lipoprotein cholesterol in patients with heterozygous familial hypercholesterolaemia on stable statin dose with or without ezetimibe therapy: a phase 2 randomised controlled trial. *The Lancet*. **380**: 29–36.

8. Bays, H., D. Gaudet, R. Weiss, J. L. Ruiz, G. F. Watts, I. Gouni-Berthold, J. Robinson, J. Zhao, C. Hanotin, and S. Donahue. 2015. Alirocumab as Add-On to Atorvastatin Versus Other Lipid Treatment Strategies: ODYSSEY OPTIONS I Randomized Trial. *The Journal of Clinical Endocrinology & Metabolism*. **100**: 3140–3148.

9. Cannon, C. P., B. Cariou, D. Blom, J. M. McKenney, C. Lorenzato, R. Pordy, U. Chaudhari, and H. M. Colhoun. 2015. Efficacy and safety of alirocumab in high cardiovascular risk patients with inadequately controlled hypercholesterolaemia on maximally tolerated doses of statins: the ODYSSEY COMBO II randomized controlled trial. *European Heart Journal*. **36**: 1186–1194.

10. Stroes, E., J. R. Guyton, N. Lepor, F. Civeira, D. Gaudet, G. F. Watts, M. T. Baccara-Dinet, G. Lecorps, G. Manvelian, M. Farnier, and the ODYSSEY CHOICE II Investigators. 2016. Efficacy and Safety of Alirocumab 150 mg Every 4 Weeks in Patients With Hypercholesterolemia Not on Statin Therapy: The ODYSSEY CHOICE II Study. *Journal of the American Heart Association*. **5**: e003421.

11. Dias, C. S., A. J. Shaywitz, S. M. Wasserman, B. P. Smith, B. Gao, D. S. Stolman, C. P. Crispino, K. V. Smirnakis, M. G. Emery, A. Colbert, J. P. Gibbs, M. W. Retter, B. P. Cooke, S. T.

Uy, M. Matson, and E. A. Stein. 2012. Effects of AMG 145 on Low-Density Lipoprotein Cholesterol Levels. *Journal of the American College of Cardiology*. **60**: 1888–1898.

12. Giugliano, R. P., N. R. Desai, P. Kohli, W. J. Rogers, R. Somaratne, F. Huang, T. Liu, S. Mohanavelu, E. B. Hoffman, S. T. McDonald, T. E. Abrahamsen, S. M. Wasserman, R. Scott, and M. S. Sabatine. 2012. Efficacy, safety, and tolerability of a monoclonal antibody to proprotein convertase subtilisin/kexin type 9 in combination with a statin in patients with hypercholesterolaemia (LAPLACE-TIMI 57): a randomised, placebo-controlled, dose-ranging, phase 2 study. *The Lancet*. **380**: 2007–2017.

13. Koren, M. J., P. Lundqvist, M. Bolognese, J. M. Neutel, M. L. Monsalvo, J. Yang, J. B. Kim, R. Scott, S. M. Wasserman, and H. Bays. 2014. Anti-PCSK9 Monotherapy for Hypercholesterolemia. *Journal of the American College of Cardiology*. **63**: 2531–2540.

14. Raal, F., R. Scott, R. Somaratne, I. Bridges, G. Li, S. M. Wasserman, and E. A. Stein. 2012. Low-Density Lipoprotein Cholesterol-Lowering Effects of AMG 145, a Monoclonal Antibody to Proprotein Convertase Subtilisin/Kexin Type 9 Serine Protease in Patients With Heterozygous Familial Hypercholesterolemia: The Reduction of LDL-C With PCSK9 Inhibition in Heterozygous Familial Hypercholesterolemia Disorder (RUTHERFORD) Randomized Trial. *Circulation*. **126**: 2408–2417.

15. Sullivan, D., A. G. Olsson, R. Scott, J. B. Kim, A. Xue, V. Gebski, S. M. Wasserman, and E. A. Stein. 2012. Effect of a Monoclonal Antibody to PCSK9 on Low-Density Lipoprotein Cholesterol Levels in Statin-Intolerant Patients: The GAUSS Randomized Trial. *JAMA*. **308**: 2497.

16. Fitzgerald, K., S. White, A. Borodovsky, B. R. Bettencourt, A. Strahs, V. Clausen, P. Wijngaard, J. D. Horton, J. Taubel, A. Brooks, C. Fernando, R. S. Kauffman, D. Kallend, A.

Vaishnaw, and A. Simon. 2017. A Highly Durable RNAi Therapeutic Inhibitor of PCSK9. *New England Journal of Medicine*. **376**: 41–51.

17. Fitzgerald, K., M. Frank-Kamenetsky, S. Shulga-Morskaya, A. Liebow, B. R. Bettencourt, J. E. Sutherland, R. M. Hutabarat, V. A. Clausen, V. Karsten, J. Cehelsky, S. V. Nochur, V. Kotelianski, J. Horton, T. Mant, J. Chiesa, J. Ritter, M. Munisamy, A. K. Vaishnaw, J. A. Gollob, and A. Simon. 2014. Effect of an RNA interference drug on the synthesis of proprotein convertase subtilisin/kexin type 9 (PCSK9) and the concentration of serum LDL cholesterol in healthy volunteers: a randomised, single-blind, placebo-controlled, phase 1 trial. *The Lancet*. **383**: 60–68.

18. Ray, K. K., U. Landmesser, L. A. Leiter, D. Kallend, R. Dufour, M. Karakas, T. Hall, R. P. T. Troquay, T. Turner, F. L. J. Visseren, P. Wijngaard, R. S. Wright, and J. J. P. Kastelein. 2017. Inclisiran in Patients at High Cardiovascular Risk with Elevated LDL Cholesterol. *New England Journal of Medicine*. **376**: 1430–1440.

19. Baruch, A., D. Luca, R. S. Kahn, K. J. Cowan, M. Leabman, N. R. Budha, C. P. C. Chiu, Y. Wu, D. Kirchhofer, A. Peterson, J. C. Davis Jr, and W. G. Tingley. 2017. A phase 1 study to evaluate the safety and LDL cholesterol-lowering effects of RG7652, a fully human monoclonal antibody against proprotein convertase subtilisin/kexin type 9: RG7652, an anti-PCSK9 antibody, reduces LDL-C. *Clinical Cardiology*. **40**: 503–511.

20. Crispell, K. R., B. Porter, and R. T. Nieset. 1950. Studies of plasma volume using human serum albumin tagged with radioactive iodine. *J. Clin. Invest.* **29**: 513–516.

21. Schulz, R., K.-D. Schlüter, and U. Laufs. 2015. Molecular and cellular function of the proprotein convertase subtilisin/kexin type 9 (PCSK9). *Basic Research in Cardiology*. **110**. [online] http://link.springer.com/10.1007/s00395-015-0463-z (Accessed April 19, 2018).

22. Langer, T., W. Strober, and R. I. Levy. 1972. The metabolism of low density lipoprotein in familial type II hyperlipoproteinemia. *J. Clin. Invest.* **51**: 1528–1536.
